# Topical administration of orbital fat-derived stem cells promotes corneal tissue regeneration

**DOI:** 10.1186/scrt223

**Published:** 2013-06-14

**Authors:** Ko-Jo Lin, Mei-Xue Loi, Gi-Shih Lien, Chieh-Feng Cheng, Hsiang-Yin Pao, Yun-Chuang Chang, Andrea Tung-Qian Ji, Jennifer Hui-Chun Ho

**Affiliations:** 1 Department of Ophthalmology, Wan Fang Hospital, Taipei Medical University, 111 Hsing-Long Road, Sec. 3, Taipei 116, Taiwan; 2 Department of Internal Medicine, Wan Fang Hospital, Taipei Medical University, 111 Hsing-Long Road, Sec. 3, Taipei 116, Taiwan; 3 Center for Stem Cell Research, Wan Fang Hospital, Taipei Medical University, 111 Hsing-Long Road, Sec. 3, Taipei 116, Taiwan; 4 Graduate Institute of Clinical Medicine, Taipei Medical University, 250 Wu-Hsing Street, Taipei 110, Taiwan

**Keywords:** Corneal injury, Inflammation, Mesenchymal stem cells, Orbital fat-derived stem cells, Topical administration

## Abstract

**Introduction:**

Topical administration of eye drops is the major route for drug delivery to the cornea. Orbital fat-derived stem cells (OFSCs) possess an *in vitro* corneal epithelial differentiation capacity. Both the safety and immunomodulatory ability of systemic OFSC transplantation were demonstrated in our previous work. In this study, we investigated the safety, therapeutic effect, and mechanism(s) of topical OFSC administration in an extensive alkali-induced corneal wound.

**Methods:**

Corneal injury was created by contact of a piece of 0.5 N NaOH-containing filter paper on the corneal surface of a male Balb/c mouse for 30 s. The area of the filter paper covered the central 70% or 100% of the corneal surface. OFSCs (2 × 10^5^) in 5 μl phosphate-buffered saline (PBS) were given by topical administration (T) twice a day or by two intralimbal (IL) injections in the right cornea, while 5 μl of PBS in the left cornea served as the control.

**Results:**

Topical OFSCs promoted corneal re-epithelialization of both the limbal-sparing and limbal-involved corneal wounds. In the first three days, topical OFSCs significantly reduced alkali-induced corneal edema and stromal infiltration according to a histopathological examination. Immunohistochemistry and immunofluorescence staining revealed that transplanted cells were easily detectable in the corneal epithelium, limbal epithelium and stroma, but only some of transplanted cells at the limbal epithelium had differentiated into cytokeratin 3-expressing cells. OFSCs did not alter neutrophil (Ly6G) levels in the cornea, but significantly reduced macrophage (CD68) infiltration and inducible nitrous oxide synthetase (iNOS) production during acute corneal injury as quantified by a Western blot analysis. Continuous topical administration of OFSCs for seven days improved corneal transparency, and this was accompanied by diffuse stromal engraftment of transplanted cells and differentiation into p63-expressing cells at the limbal area. The therapeutic effect of the topical administration of OFSCs was superior to that of the IL injection. OFSCs from the IL injection clustered in the limbal area and central corneal epithelium, which was associated with a persistent corneal haze.

**Conclusions:**

Topical OFSC administration is a simple, non-surgical route for stem cell delivery to promote corneal tissue regeneration through ameliorating acute inflammation and corneal epithelial differentiation. The limbal area serves as a niche for OFSCs differentiating into corneal epithelial cells in the first week, while the stroma is a potential site for anti-inflammation of OFSCs. Inhibition of corneal inflammation is related to corneal transparency.

## Introduction

Both a limbal cell deficiency and corneal stromal injury affect long-term corneal transparency due to corneal opacity [1]. During corneal tissue injury, inflammation is harmful to epithelial cell migration, and inflammatory cell infiltration in the stroma aggravates corneal edema and scarring [2]. The cornea is physiologically re-epithelialized by the proliferation and differentiation of limbal stem cells in the basal layer of the limbal epithelium [3,4], and extensive loss of limbal epithelial cells leads to a persistent corneal epithelial defect and pannus formation [5]. Damage to the corneal stroma represents corneal ulceration, and this is complicated by corneal opacity due to scar formation [6].

Corneal transplantation is a way to replace scar tissue using the full or partial thickness of a donor’s central cornea [7], but it fails to regenerate limbal stem cells. Moreover, the donor source and graft rejection are major limitations of corneal transplantations [8]. Limbal transplantation is a surgical procedure that removes autologous limbal epithelium from the contralateral eye to replenish diseased limbal epithelium [9]; disruption of healthy limbal stem cells is inevitable. Recently, replacement of the corneal epithelium under a limbal cell deficiency is achieved by *ex vivo* cultured cells, including limbal stem cells [10,11], conjunctival epithelial cells [12], and oral mucosal cells [13]. However, long-term graft survival is always a challenge with autologous conjunctival or oral epithelial cell transplantation due to a lack of stem cell properties of those cells. Stem cell transplantation is a new therapeutic strategy for corneal tissue regeneration that relies on their multipotency.

In terms of stem cell therapy, healthy limbal stem cell preservation and immune tolerance of stem cells are two critical issues for successful corneal regeneration [14,15], and it is imperative to use immune-tolerant allogenic stem cells. Among stem cells, only mesenchymal stem cells (MSCs) possess the immunomodulatory ability and are well-tolerated during allogenic transplantation [16]. We have successfully isolated and purified multipotent stem cells from human orbital fatty tissues [17]. Orbital fat-derived stem cells (OFSCs) are MSCs isolated from human orbital fat tissue [18]. In our previous study, we have demonstrated that the growth kinetics of OFSCs is similar to bone marrow-derived MSCs (BM-MSCs), while more than 260 surface markers of OFSCs are consistent with BM-MSCs [17,19,20]. OFSCs lack immunogenecity, and the safety and immunomodulatory ability of systemic OFSC transplantation has been demonstrated in our previous xenotransplant model [20]. In addition, OFSCs possess the osteogenic, chondrogenic and adipogenic differentiation capacity, and may differentiate into corneal epithelial cells upon contact with human corneal epithelial cells *in vitro*[17]. Therefore, development of a technically non-surgical, non-invasive method to deliver OFSCs and allow OFSCs to directly contact corneal epithelial cells will be valuable for corneal tissue regeneration.

In this study, extensive corneal injury was created by NaOH, an alkali chemical reagent. Alkali-induced corneal injury leads to corneal/limbal epithelial defects, and induces further stromal tissue melting and severe corneal inflammation, which subsequently results in corneal edema, ulceration in the early stage, and corneal opacity/scarring in the late stage [21]. We created two sizes of corneal wounds, that is, one was a limbal-sparing central corneal injury and the other was total corneal damage, including the limbal area. OFSCs were topically administrated to the corneal surface, and the therapeutic effect was evaluated by the size of the corneal defect, the corneal thickness, stromal infiltration and corneal transparency. In this experiment, the mechanism and the niche of OFSCs before and after corneal re-epithelialization were explored. In addition, differences in limbal-involved corneal injury between topical OFSCs and an intralimbal injection of OFSCs were also compared.

## Material and methods

### Animals with the corneal injury

Male Balb/c mice were purchased from BioLASCO (Taipei, Taiwan). Animals were maintained in the animal facility of Wan Fang Hospital, Taipei Medical University (WFH-TMU, Taipei, Taiwan). All experimental protocols were approved by the animal use and care committee of WFH-TMU. An eight-week-old mouse’s cornea was covered by round filter paper, which had been rinsed by 0.5 N NaOH before covering the mouse corneal surface for 30 s. Mice were separated into two groups: filter paper in group A covered 70% of the central corneal area (5.9 mm in diameter) and in group B, filter paper covered 100% of the corneal area (7 mm in diameter). Corneal epithelial cells were smoothly removed with a no. 15 Bard-Parker scalpel blade after alkali damage.

### Isolation, expansion of OFSCs

Isolation and culture of OFSCs were carried out as described previously [17]. Briefly, during blepharoplastic surgery, 0.5 to approximately 1 ml of redundant orbital fat tissues was removed from the intraorbital cavity. All samples were removed with informed consent and followed regulations of the Institutional Review Board of WFH-TMU. Tissues were fragmented, digested and filtered. The suspension was centrifuged, cells from the pellet were plated at a very low density, and colony-forming cells were maintained in MesenPro medium (Invitrogen, Carlsbad, CA, USA). OFSCs were mesenchymal stem cells which were negative for CD34, CD133, CD31, CD106, CD146, CD45, CD14, CD117 and HLA-DR and positive for CD58, CD90, CD105, CD29, CD49b, CD49e, CD44, CD49d and HLA-ABC [17]. The tri-lineage differentiation capacity of these cells was checked before this study.

### Topical and intra-limbal OFSC transplantation

Six mice were used in Group A. After 70% of corneal injury to both eyes, the right eye was applied with topical OFSCs (T) and the left eye with PBS. Night mice were used in Group B for the topical (T) administration of OFSCs. After 100% of corneal injury for both eyes took place, topical OFSCs were given to the right eyes while topical PBS (six mice) or no treatment (dry control, three mice) to the left eyes. In addition, three mice without corneal injury received topical OFSCs on the right eyes and topical PBS on the left eyes.

For evaluation of the intra-limbal (IL) injection, 100% corneal injury was created on three mouse corneas. IL injection of OFSCs was performed in the right eye and PBS in the left eye. One mouse without corneal injury received IL OFSCs in the right eye and IL PBS in the left eye.

Before treatment, OFSCs were detached and re-suspended in PBS(Gibco, Grand Island, NY, USA). For topical (T) administration, 2 × 10^5^ human OFSCs with or without quantum dots (Invitrogen) labeling in 5 μl of PBS were applied to the right corneal surface twice a day until the day of sacrifice, while 5 μl of PBS applied to the left eye twice a day served as the control. For the IL injection, 2 × 10^5^ quantum dots (Invitrogen)-labeled OFSCs in 5 μl of PBS were injected into the lateral side of the right limbal epithelium on the first day, and a repeat injection on the nasal side of the right limbal epithelium was given on Day 6, while 5 μl of PBS was injected into the left limbal epithelium at the same time.

### Quantification the area of corneal injury

The cornea wound was examined on days 0, 1, 2, 3 and 7. Before being photographed, the epithelial defect was stained with a topical fluorescent strip (HAAG-STREIT, Koeniz, Switzerland), and images were captured with a digital camera (Canon, Tokyo, Japan) under a cobalt-blue light source from a direct ophthalmoscope (Welch Allyn, Skaneateles Falls, NY, USA). The injured area of the cornea was determined using the software Image Pro-Plus version 6.0 (Media Cybernetics, Rockville, MD, USA) and calculated as a percentage of the residual epithelial defect.

### Histological and immunohistochemical (IHC) staining

Mice were sacrificed at the end of day 2, 3 or 7 after injury. The eyeball was removed *en bloc* and fixed in formalin, then prepared in paraffin-embedded blocks for sectioning at a thickness of 10 μm. Tissue sections were stained with hematoxylin and eosin (H&E) (Sigma-Aldrich, St. Louis, MO, USA). For IHC staining, tissue sections were incubated with rabbit antibody against human immunoglobulin G (hIgG) (1:800, Abcam, Cambridge, MA, USA), or rabbit antibody against human beta-2 microglobulin (hβ2M) (1:800, Abcam) at 4°C for 1 h, followed by goat antibodies against rabbit IgG (Dako Cytomation, Glostrup, Denmark) for another 40 to approximately 60 minutes. Tissue sections were assessed by microscopy (Leica Microsystem, Wetzlar, Germany). Images were acquired with MetaMorph version 4.6 (Molecular Devices, Sunnyvale, CA, USA).

### Immunofluorescence staining

For cytokeratin 19 (CK19) and CK3 staining, frozen section tissue slides were fixed in cold methanol for 30 minutes, followed by two PBS washes. After being blocked in 5% skim milk at room temperature for 1 h, slides were incubated with a mouse antibody against human/mouse CK19 (1:500, Millipore, Billerica, MA, USA), a mouse antibody against human/mouse CK3 (1:500, Millipore), rabbit antibody against human p63 (1:150, Abcam), or rabbit antibody against hIgG (1:800, Abcam) at room temperature for 1 h, followed by incubation with DyLight 488-conjugated goat anti-mouse IgG (1:500, Jackson ImmunoResearch Laboratories, Inc. West Grove, PA, USA), or DyLight 594-conjugated goat anti-rabbit IgG (1:500, Jackson ImmunoResearch Laboratories, Inc.) at room temperature for 30 minutes. Nuclei were then stained with 4,6-diamidino-2-phenylindole (DAPI, 1:1,000), and samples were assessed under a fluorescence microscope (Leica Microsystem). Images were acquired using MetaMorph version 4.6 (Molecular Devices).

### Western blot analysis

Total protein was obtained from mouse cornea with Cell Lysis Buffer (Sigma-Aldrich) containing freshly added protease inhibitors (Sigma-Aldrich). Protein concentrations were determined with a Bio-Rad Protein Assay (Bio-Rad Laboratories, Hercules, CA, USA). Electrophoresis was performed using 30 μg of total protein by 10% sodium dodecylsulfate polyacrylamide gel electrophoresis (SDS-PAGE) and transferred to polyvinylidenedifluoride membranes (Millipore). Nonspecific binding was blocked by 5% skim milk in TBST buffer (50 mMTris–HCl, pH 7.4; 150 mMNaCl, and 0.1% Tween 20) at room temperature for 1 h. The membrane was then probed with a rabbit polyclonal antibody (pAb) to inducible nitrous oxide synthetase (iNOS, 1:2000, Abcam), a rabbit monoclonal antibody (mAb) to CD68 (1:1,000, Epitomics, Burlingame, CA, USA), a rabbit pAb to tissue growth factor-beta (TGF-β, 1:4,000, Abcam), a mouse mAb to tumor necrosis factor-alpha (TNF-α, 1:1,000, Abcam), a rat mAb to lymphocyte antigen 6 complex (Ly6G, 1:1,000, Abcam), a mouse mAb to vascular endothelial growth factor (VEGF, 1:1,000, Abcam), a rabbit pAb to hIgG (1:800, Abcam), a mouse mAb to mouse and human β-actin (1:200, Sigma-Aldrich), or a mouse mAb to α-tubulin antibodies (1:10^4^, Sigma-Aldrich) at 4°C overnight. After three washes with TBST (for 15 minutes each), the membrane was incubated with a horseradish peroxidase (HRP)-conjugated secondary antibody against to rabbit IgG (1:5,000, Santa Cruz Biotechnology, Santa Cruz, CA, USA) or against to mouse IgG (1:5,000, Abcam) at room temperature for 1 h. After three more TBST washes, protein signals were detected by enhanced chemiluminescence (ECL; NEN Life Science, Boston, MA, USA) and their intensities were measured by densitometry (Image Pro-Plus version 6.0, Media Cybernetics).

### Statistical analysis

Values are shown as the mean ± standard error. Statistical analyses were performed using the Statistical Package for Social Science version 16 software (SPSS, Chicago, IL, USA). Results of comparisons of the area of corneal injury at each time point between the PBS and OFSCs groups or between the dry control and OFSCs groups, and corneal protein expression between the PBS and OFSCs groups were analyzed by Student’s *t*-test, and *P <*0*.*05 was considered a statistically significant difference.

## Results

### OFSCs promote corneal wound healing

Mice were divided into two groups. A 70% central corneal injury (limbal-sparing) was created in group A, and 100% corneal damage (limbal-involved) was created in group B. Topical 2 × 10^5^ OFSCs in 5 μl PBS (OFSCs-(T)) was administrated to the right corneal surface twice a day, and topical 5 μl PBS (PBS-(T)) was given to the left cornea twice a day.

**Figure 1:**
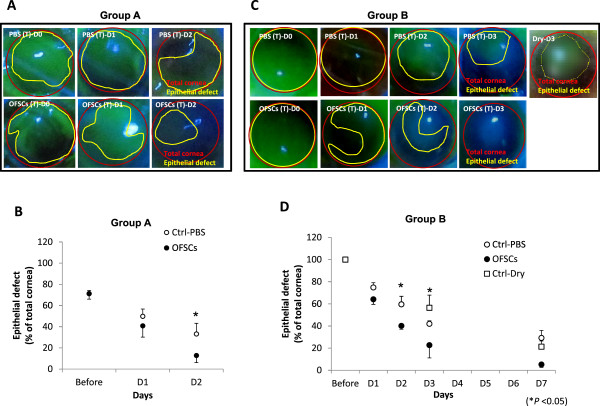
**Orbital fat-derived stem cells (OFSCs) promoted corneal wound healing.** In group A, a 70% central corneal defect was created. Topical (T) administration of OFSCs promoted corneal wound healing (**A**) and the area of residual epithelial defect under OFSCs was significantly lower than that of the PBS group on Day 2 (**B**). In group B, a 100% corneal injury with limbal involvement was created. The injured area significantly decreased with topical OFSC treatment. OFSCs promoted corneal re-epithelialization (**C**), and the injured area significantly decreased with topical OFSCs after the second day (**D**). (*t*-test, * *P* <0.05).

For the limbal-sparing corneal wound (group A), OFSCs promoted corneal re-epithelialization (Figure 1A). Areas of the epithelial defect after alkali-injury were 71.3% ± 3.03% of the total cornea, 49.8% ± 6.87% on Day 1, and 33.2% ± 10.06% on Day 2, while they were 71.2% ± 5.13% after injury, 40.8% ± 10.64% on Day 1, and 12.8% ± 6.77% on Day 2 with topical OFSC treatment (Figure 1B).

For limbal-involved corneal wounds (group B), OFSCs accelerated corneal wound healing but a central corneal haze was found in the first three days (Figure 1C). In mice with a 100% corneal epithelial defect after alkali-injury, areas of the epithelial defect under PBS treatment were 74.8% ± 4.32% of the total cornea on Day 1, 59.6% ± 9.37% on Day 2, 42.0% ± 2.74% on Day 3, and 29.3% ± 6.78% on Day 7; while they were 64.2% ± 4.59% on Day 1, 40.2% ± 3.06% on Day 2, 15.0% ± 7.83% on Day 3, and 7.9% ± 2.34% on Day 7 with topical OFSC treatment (Figure 1D). Compared with PBS control, the dry control on a 100% corneal injury showed no significant difference on both corneal haze (Figure 1B, Dry-D3) and residual corneal defect (Figure 1D) in the first seven days.

### Topical OFSCs’ initial contact with the corneal epithelium, limbal epithelium, and stroma

**Figure 2:**
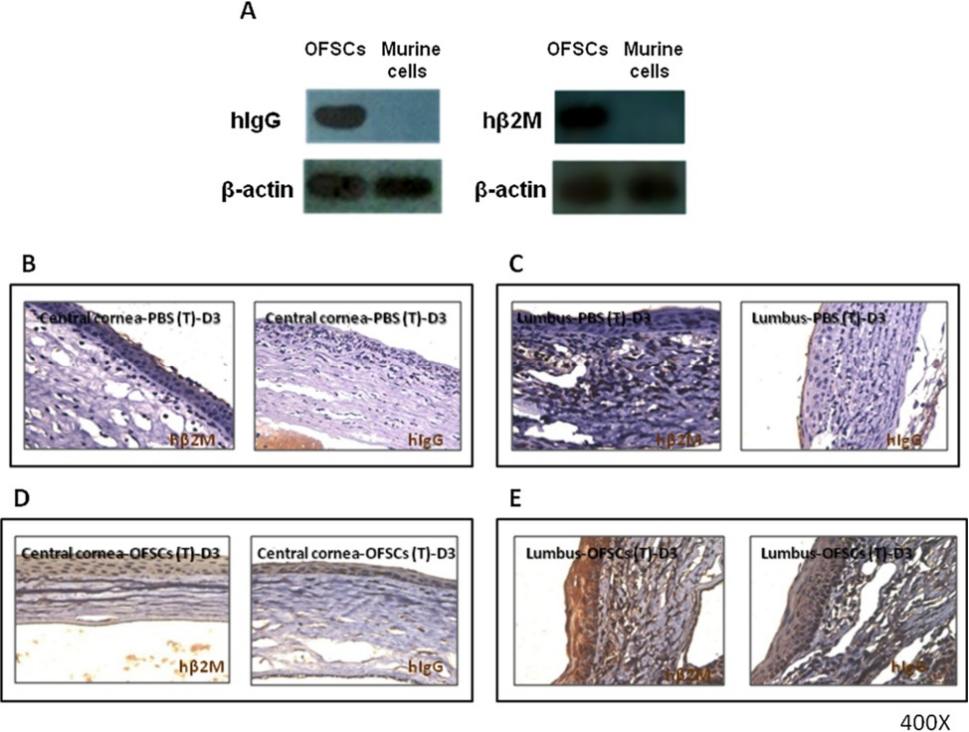
**Topical OFSCs were initially engrafted into the corneal epithelium, limbal epithelium and stroma.** OFSCs, different from murine cells, expressed human immunoglobulin (hIgG) and human β-2 microglobulin (hβ2M) (**A**). Two human-specific proteins, hβ2M and hIgG, were not found in the PBS-treated central cornea (**B**) and limbal area (**C**). In the limbal-involved corneal wound, topical OFSCs were detected in the central epithelium (**D**), limbal epithelium (**E**) and stroma area (**D**, **E**).

OFSCs, isolated from human orbital fat tissues, may be distinguished from murine cells by targeting human-specific housekeeper proteins, i.e. hIgG (Figure 2A, left) and hβ2M (Figure 2A, right). To identify the distribution of transplanted cells, hβ2M and hIgG were stained on corneal sections. In PBS-treated eyes, no human cells were detectable in either the central cornea (Figure 2B) or limbal area (Figure 2C). In OFSC-treated eyes, hβ2M and hIgG-expressing cells were found in the central corneal epithelium (Figure 2D), limbal epithelium (Figure 2E) and stroma (Figure 2D,E).

### Initial corneal epithelial differentiation of OFSCs is observed in the limbal epithelium

**Figure 3:**
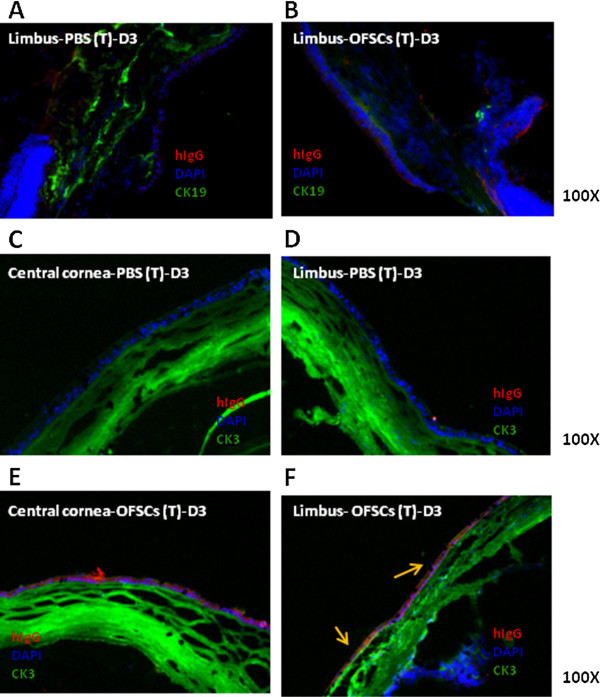
**Initial corneal epithelial differentiation of OFSCs was observed in the limbal epithelium.** (**A**) Cytokeratin (CK) 19 was primarily stained in the limbal area, but neither human immunoglobulin G (hIgG) nor CK19 co-expressing cells were found in the limbal area after topical OFSC treatment of the limbal-damaged cornea for three days (**B**). CK3 expression was noted in both the central (**C, E**) and limbal (**D, F**) cornea. Three days after topical OFSC treatment, some hIgG and CK3 co-expressing cells were observed in the limbal epithelium (**F**), but not in the (**D**) central epithelium.

We further determined whether transplanted OFSCs differentiated into corneal epithelial cells within three days. Using immunofluorescence staining, CK19 was stained in the limbal cornea (Figure 3A, B) as a libal progenitor marker [22,23], and no human cells expressing CK19 were found (Figure 3B). Although OFSCs were found in the corneal epithelium, limbal epithelium and stroma (Figure 2C,D), no OFSCs differentiated into CK3-expressing cells, which is a marker of a well-differentiated cornea, in the central cornea (Figure 3E). Some transplanted cells co-expressing CK3 were found in the limbal epithelium (Figure 3F, yellow arrows).

### OFSCs reduce alkali-induced acute inflammation and corneal edema

**Figure 4:**
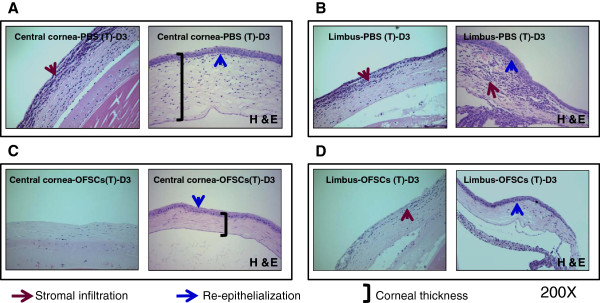
**OFSCs reduced alkali-induced inflammation and corneal edema.** An alkali reagent induced a large corneal epithelial defect with severe stromal infiltration and corneal edema in the first three days, which were found in the central cornea (**A**) and limbal area (**B**). OFSCs promoted corneal re-epithelialization, decreased stromal infiltration and reduced corneal thickness in the central cornea (**C**) and limbal area (**D**).

During the first three days, severe inflammatory cell infiltration and increases in the corneal thickness were found in the stroma of the central cornea (Figure 4A, brown arrow) and limbal area (Figure 4B, brown arrow); this was (Figure 4A, left, 4B, left) or was not (Figure 4A, right, 4B, right, blue arrow) independent of the epithelial defect. OFSCs significantly ameliorated alkali-induced stromal infiltration, and this was also independent of corneal re-epithelialization (Figure 4C,D). In the area of re-epithelialization (Figure 4C, right, 4D, right, blue arrow), OFSCs further reduced the corneal thickness, especially in the central area (Figure 4C, right).

### OFSCs inhibit macrophage infiltration and iNOS production during acute corneal inflammation

**Figure 5:**
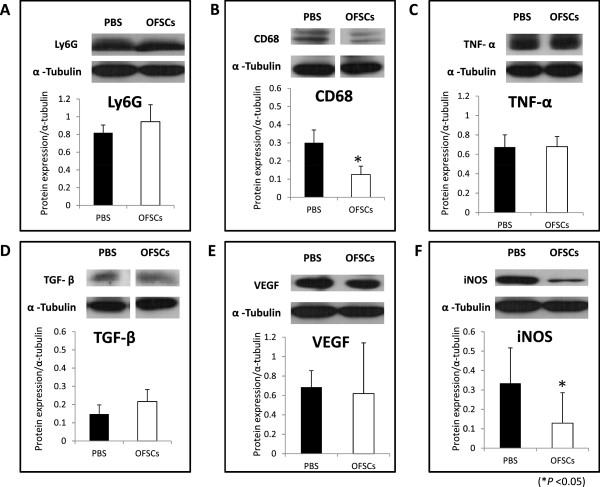
**OFSCs inhibited macrophage infiltration and inducible nitric oxide synthase (iNOS) production in the cornea.** A Western blot analysis and its quantification demonstrated that OFSCs did not alter the expression of lymphocyte antigen 6 complex (Ly6G) (**A**) but significantly decreased the CD68 (**B**) level in the cornea after alkali injury. Measurements of macrophage-related proinflammatory cytokine levels in the alkali-damaged cornea revealed that OFSCs had no effect on regulating tumor necrosis factor (TNF)-α (**C**), transforming growth factor (TGF)-β (**D**), or vascular endothelial growth factor (VEGF) (**E**), but significantly reduced iNOS (**F**). α-Tubulin served as the internal control. (*t*-test, * *P* <0.05).

Both macrophage and neutrophil infiltration occurred in response to acute tissue injury. We performed a Western blot analysis to measure the differential expression level of Ly6G, a neutrophil marker [24], and CD68, a macrophage marker [25], between PBS- and OFSC-treated corneas three days after alkali injury. It was demonstrated that OFSCs did not alter Ly6G protein expression (Figure 5A) but significantly decreased the CD68 level (Figure 5B). Furthermore, macrophage-produced iNOS and several macrophage-released cytokines, such as TGF-β, TNF-α and VEGF [26-29], were reported to enhance corneal inflammation, neovascularization and persistence of epithelial defects. We found that OFSCs had no effect on TNF-α (Figure 5C), TGF-β (Figure 5D) or VEGF (Figure 5F) protein expressions, but significantly reduced iNOS production (Figure 5G).

### Intra-limbal injection of OFSCs is not sufficient to clear the cornea

**Figure 6:**
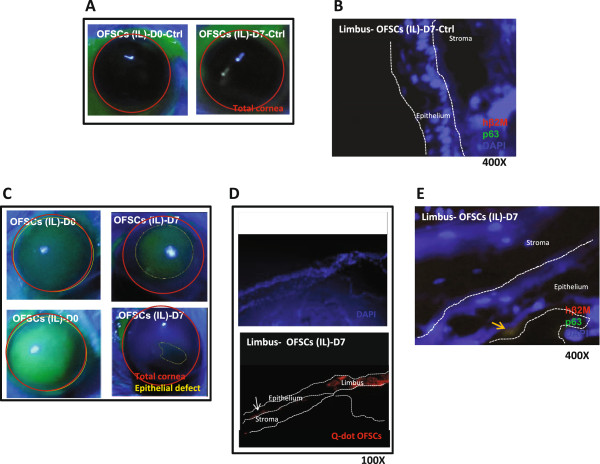
**Intra-limbal injection of OFSCs was not a proper route for corneal regeneration.** For an intact cornea, repeated intra-limbal (IL) injection of OFSCs was safe (**A**) but no p63-expressing cells were differentiated from transplanted cells (**B**). For an extensively damaged cornea, corneal opacity was obvious after repeated IL OFSC injections (**C**). Quantum dot-labeled transplanted cells were clustered at the injection site, and some were detectable in the central corneal epithelium (**D**). Only a few OFSCs differentiated into p63-expressing cells at the limbal area seven days after corneal injury (**E**).

**Figure 7:**
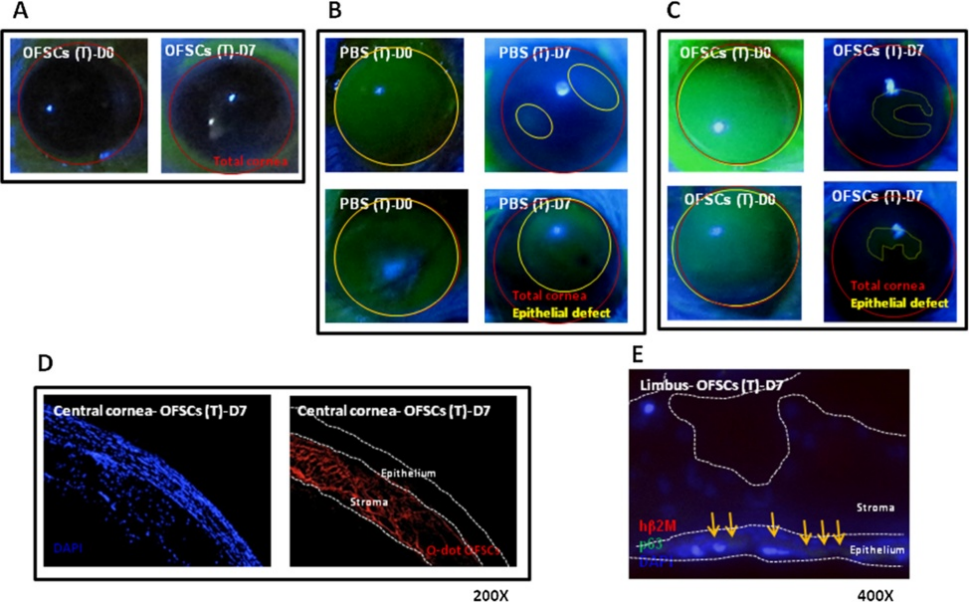
**Diffuse stromal engraftment of OFSCs contributed to the corneal transparency.** Seven days after multiple rounds of topical administration (**A**), OFSCs had not harmed the cornea. For the limbal-involved corneal injury, topical OFSCs (**C**) showed better therapeutic effects on wound healing and corneal transparency than topical PBS (**B**). Most quantum dot-labeled OFSCs were diffusely distributed in the stroma (**D**), while some of transplanted cells differentiated into p63-expressing cells at limbal epithelium (**E**) seven days after topical OFSC administration.

The limbal area served as a place for corneal epithelial cell differentiation (Figure 3F). We further sent quantum dots-labeled OFSCs into the corneal area by intra-limbal (IL) injection and avoided OFSCs contacting the corneal stroma. As shown in Figure 6, repeated IL injection of OFSCs in an intact cornea did not alter corneal transparency (Figure 6A). However, neither hβ2M nor human p63 expressing cells could be found at the injection site (Figure 6B). The therapeutic effect of IL OFSC injection in a damaged cornea with limbal involvement (Figure 6C) was not compatible with topical OFSC administration (Figure 7C) in the first seven days. We found that some OFSCs clustered at the injection site (Figure 6D), and some were located in the central corneal epithelium (Figure 6D, white arrow), but only a few of the injected cells differentiated into p63, a human limbal progenitor marker, positive cells.

### Stromal engraftment of topical OFSCs contributes to corneal transparency

Topical application of OFSCs in the first three days was insufficient to improve corneal transparency in the limbal-involved corneal wound (Figure 1C), so the long-term effect of OFSCs needed to be investigated. For safety consideration, multiple rounds of topical OFSCs on the normal corneal surface did not attack the corneal epithelium (Figure 7A). In the limbal-involved corneal wound, a large corneal epithelial defect and marked corneal opacity were observed under PBS treatment for seven days (Figure 7B), and continuous topical OFSC administration for seven days reduced the wound size and improved the corneal transparency (Figure 7C). Fluorescent staining revealed that diffuse quantum dot-labeled OFSCs were found in the corneal stroma, but no longer in the central corneal epithelium (Figure 7D). Limbal sections showed that some of the hβ2M-expressing cells differentiated into p63 positive cells (Figure 7E).

## Discussion

For the first time, we proved the concept that multiple rounds of topical OFSCs is a simple, non-surgical strategy to promote corneal tissue regeneration. In the first three days, the therapeutic effects of topical OFSCs are mainly through inflammation inhibition (Figures 1 and 4), but little by directional corneal epithelial cell differentiation (Figure 3). Inhibition of macrophage infiltration and iNOS production (Figure 5) accounts for the amelioration of acute corneal inflammation and promotion of corneal re-epithelialization. OFSCs remain in the limbal epithelium, corneal epithelium and stroma in the first three days (Figure 2), and the limbal epithelium is favored for corneal epithelial differentiation (Figures 3, 6E and 7E). However, the corneal stroma potentially serves as a niche for OFSC engraftment, and this is related to improvements in the corneal transparency (Figures 6 and 7).

OFSCs, the same as BM-MSCs, lacked immunogenicity and were well-tolerated after intravenous injections in our previous studies [20,30]. This time, we explored whether topical administration (Figure 7A) and IL injections (Figure 6A) were safe routes to deliver MSCs into corneal tissues. Clinically, the addition of topical steroids or non-steroid anti-inflammatory drugs benefits corneal re-epithelialization and prevents corneal scar formation [31,32]. Systemic transplantation of a high dose of MSCs reduce ethanol-induced inflammatory damage to the cornea by secretion of TNF-α-stimulated gene/protein 6 with minimal engraftment [33], which shows that systemic administration is not an efficient way to deliver stem cells to the cornea, a physiologically avascular tissue. However, the abundant paracrine effect of MSCs contributes to inhibition of inflammation. It has been reported that BM-MSCs expanded on an amniotic membrane can reconstruct an alkali-damaged cornea by inhibiting inflammation and angiogenesis [34]. Alternatively, topical administration of stem cells provided stem cells with direct contact with the corneal epithelium and stroma on a damaged cornea (Figures 2 and 7D), and low-dose OFSCs resulted in good corneal protection (Figures 1, 3, 4 and 7).

Neutrophils and macrophages, known as immune cells, are responsible for innate immunity, and infiltrate into an acutely inflamed cornea [35]. Macrophages play the central role in acute inflammation because they produce various proinflammatory cytokines, and also recruit and activate T-lymphocytes [35,36]. Macrophage-produced iNOS and VEGF cause a vicious cycle with alkali-induced corneal inflammation [26,36]. According to our data, OFSCs inhibited macrophage infiltration and subsequent iNOS production in the cornea (Figure 5B,F) without altering VEGF production (Figure 5E), indicating that macrophages are target cells regulated by OFSCs during acute inflammation. Further studies on interactions between OFSCs and macrophages in regulating acute tissue inflammation are ongoing in our lab.

In our previous study, we found that cell-cell interactions between corneal epithelial cells and stem cells were crucial for corneal epithelial cell differentiation of OFSCs [17]. When we applied OFSCs to the corneal surface with a large epithelial defect, most of the transplanted cells remained in the limbal epithelium, corneal epithelium and stroma during the first few days (Figure 2). Notably, only some of the OFSCs in the limbal epithelium rapidly differentiated into CK3-expressing cells, but no CK19 signals in transplanted cells were found in the limbal area in the first three days (Figure 3), while human p63-expressing cells were detectable at limbal area seven days later (Figures 6E and 7E) rather than in the first three days (data not shown), implying that the limbal environment possibly induces OFSC differentiation and transdifferentiation into a corneal epithelial linage. The underlying mechanism should be determined.

Wound healing is essential for tissue regeneration. However, the cornea is the window of the eye, and the transparency of the cornea determines visual acuity. Corneal tissues are physiologically composed of the corneal epithelium, stroma and endothelium. Clarity of the cornea depends on an intact corneal epithelium, tight packing of epithelial cells, constant water content, and regular arrangement of keratocytes and keratocyte-produced extracellular matrix in the stroma [1,37,38]. In this study, corneal opacity was not a complication of the limbal-sparing corneal injury (Figure 1A). When OFSCs were applied to the limbal-involved corneal wound, marked central opacity was observed on Day 3 (Figure 1C), and a clear cornea did not occur until Day 7 (Figure 7C), suggesting that improved corneal clarity occurs after re-epithelialization.

We further delivered OFSCs into the limbal epithelium of a damaged cornea to avoid OFSCs directly contacting the corneal stroma. It was shown that cells transplanted via an IL injection clustered at the injection site and in the central corneal epithelium (Figure 6D), and this was associated with marked corneal opacity (Figure 6C, right). After seven days of continuous topical OFSC administration, transplanted cells were only detectable in the stroma and no longer in the corneal epithelium (Figure 7D), illustrating that stromal engraftment of OFSCs contributes to corneal transparency. It has been reported that an intrastromal injection of MSCs significantly increased the corneal thickness and transparency and lowered light scattering in keratocyte-dysfunctional mice by their similar phenotype with keratocytes and expression of keratocyte-unique keratan-sulfated keratocan and lumican after transplantation [39]. Recently, Agorogiannis *et al*. report a case using 3 × 10^6^ autologous MSCs topically applied to the bottom of a corneal ulcer for a persistent sterile corneal epithelial defect [40]. The corneal stroma is connective tissue maintained by keratocytes, which are quiescent mesenchymal cells of neural crest origin [41], and the embryonic origins of keratocytes and orbital fat tissues are the same. It is speculated that the stroma serves as a niche for long-term OFSC engraftment. In addition, topical administration of OFSCs to the cornea with an epithelial defect provides a diffuse distribution of OFSCs in the stroma, and which favors direct OFSC-keratocyte interactions in comparison with an intrastromal injection. Future studies on developing bioactive stem cell eye drops are ongoing in our lab.

## Conclusions

Topical administration of allogenic OFSCs is a simple, non-invasive method of delivering stem cells for corneal tissue regeneration. Inflammatory inhibition and corneal epithelial differentiation by OFSCs are responsible for corneal wound healing in the first few days, and corneal stroma engraftment of OFSCs at a late stage is associated with corneal transparency.

## Abbreviations

β2M: Beta-2 microglobulin; BM-MSCs: Bone marrow-derived mesenchymal stem cells; CK: Cytokeratin; DAPI: 4,6-diamidino-2-phenylindole; ECL: Enhanced chemiluminescence; HRP: Horseradish peroxidase; IgG: Immunoglobulin G; IL: Intralimbal; iNOS: Inducible nitrous oxide synthetase; Ly6G: Lymphocyte antigen 6 complex; MSCs: Mesenchymal stem cells; OFSCs: Orbital fat-derived stem cells; PBS: Phosphate-buffered saline; T: Topical; TBST: mixture of Tris-buffered saline and Tween 20; TGF-β: Transforming growth factor-beta; TNF-α: Tumor necrosis factor-alpha; VEGF: Vascular endothelial growth factor.

## Competing interests

The authors declare that they have no competing interests.

## Authors’ contributions

KJL and MXL participated in the design of the study and the animal study and helped draft the manuscript. CFC participated in the animal study. HYP carried out the immunostaining and statistical analysis, YCC performed the Western blot analysis, and ATJ carried out fluorescence staining. GSL proofread the manuscript. JHH participated in the design of the study and drafted the manuscript. All authors read and approved the final manuscript.

## Authors’ information

KJL and MXL are chief residents in the Department of Ophthalmology, Wan Fang Hospital, Taipei Medical University. GSL is the Vice Superintendent of Wan Fang Hospital and Associate Professor in Department of Internal Medicine, Taipei Medical University. CFC is a Visiting Staff member in the Department of Ophthalmology, Wan Fang Hospital, Taipei Medical University. HYP, YCC and ATJ are research assistants in the Center for Stem Cell Research, Wan Fang Hospital, Taipei Medical University. JHH is the Director of the Center for Stem Cell Research and a Consultant Ophthalmologist in the Department of Ophthalmology, Wan Fang Hospital, Taipei Medical University. JHH is also an Associate Professor in Graduate Institute of Clinical Medicine, Taipei Medical University.
